# Disease Subtype and Procedural Determinants of Leukapheresis Efficacy: A Retrospective Single-Center Study

**DOI:** 10.3390/jcm15166235

**Published:** 2026-08-12

**Authors:** Sinan Mersin, Pelin Aytan, Seher Yontar, Mahmut Bakır Koyuncu, Aycan Erkenekli, Esin Oğuz Kozan, Enver Tekin, Eyüp Naci Tiftik

**Affiliations:** 1 Division of Hematology, Department of Internal Medicine, Faculty of Medicine, Mersin University, 33110 Mersin, Türkiyemahmutkoyuncu@mersin.edu.tr (M.B.K.);; 2 Apheresis Unit, Mersin University Hospital, 33110 Mersin, Türkiye; 3 Department of Hematology, Adıyaman Training and Research Hospital, 02200 Adıyaman, Türkiye

**Keywords:** leukapheresis, hyperleukocytosis, leukemia, apheresis efficacy, leukostasis, cytoreduction

## Abstract

**Background/Objectives**: Therapeutic leukapheresis is widely used to manage hyperleukocytosis in hematologic malignancies, yet the factors determining its efficacy remain incompletely defined. We aimed to identify clinical, biochemical, and procedural determinants of leukapheresis efficacy in a single-center cohort. **Methods**: We retrospectively analyzed 125 leukapheresis procedures performed in adult patients with hematologic malignancies between March 2012 and June 2026. At our institution, emergency leukapheresis is performed before the initiation of cytoreductive therapy, so all first procedures were treatment-naïve (repeat procedures could follow cytoreductive therapy). The primary endpoint was the white blood cell (WBC) reduction rate (%). Secondary endpoints were ≥30% and ≥50% reduction. Predictors of reduction were identified using univariate tests and multivariable regression, with within-patient correlation accounted for using cluster-robust standard errors, and pre- versus post-procedure biochemical changes were assessed for paired samples. **Results**: The median WBC reduction was 47.9%; ≥30% and ≥50% reduction were achieved in 75% and 42% of procedures, respectively. Reduction differed significantly by diagnosis (*p* < 0.001), being highest in ALL (57.4%) and lowest in CML (18.6%). In multivariable analysis, processed volume (β = 0.004, *p* < 0.001), inlet rate (β = −0.43, *p* < 0.001), acute leukemia (β = 10.4, *p* = 0.040), and baseline platelet count (β = −0.03, *p* = 0.036) were independent predictors. Leukapheresis significantly reduced LDH and uric acid levels (both *p* < 0.001). **Conclusions**: Leukapheresis efficacy is determined by disease subtype and modifiable procedural parameters. Higher processed volume and acute leukemia diagnosis were associated with greater WBC reduction, while CML blast crisis responds poorly. These findings support procedure optimization to maximize cytoreduction.

## 1. Introduction

Hyperleukocytosis is a hematologic emergency most frequently encountered in acute leukemias and chronic myeloid leukemia (CML) in blast crisis [1,2]. Although it is often defined as a WBC count exceeding 100 × 10^9^/L, this threshold is largely arbitrary and varies by disease type; leukostasis may occur at lower counts in acute myeloid leukemia, whereas considerably higher counts are frequently tolerated in lymphoid and chronic leukemias [2,3]. The accumulation of poorly deformable leukemic blasts within the microcirculation can precipitate tissue hypoxia and organ dysfunction, manifesting as respiratory failure, neurological compromise, and a markedly elevated risk of early mortality [3,4]. Among patients presenting with hyperleukocytosis, early death rates of 20–40% have been reported [3,5], underscoring the need for prompt cytoreductive intervention.

Therapeutic leukapheresis offers a means of rapidly lowering the circulating leukocyte burden, thereby reducing the immediate risk of leukostatic complications while definitive chemotherapy is initiated [6]. Although the procedure is incorporated into clinical practice and addressed in apheresis guidelines [7], its role remains debated [8], in part because the determinants of its efficacy are not well characterized. Reported reductions in WBC count vary considerably across studies [9], and the relative contributions of disease biology, baseline laboratory parameters, and procedural variables to this variability have not been systematically clarified.

These knowledge gaps are particularly evident in three areas. Few studies have examined whether efficacy differs across hematologic malignancy subtypes [10], or whether modifiable technical parameters—such as processed blood volume, inlet flow rate, and vascular access—influence the magnitude of cytoreduction [11]. The impact of leukapheresis on biochemical markers of tumor burden, including lactate dehydrogenase (LDH) and uric acid, has likewise received limited attention [12].

To address these gaps, we conducted a retrospective analysis of leukapheresis procedures performed at our institution over a period spanning 2012 to 2026. We sought to characterize the efficacy of leukapheresis and to identify the clinical, biochemical, and procedural factors associated with the degree of leukocyte reduction, with the aim of informing procedure optimization.

## 2. Materials and Methods

### 2.1. Study Design and Population

This single-center, retrospective cohort study was conducted at the Division of Hematology, Mersin University Faculty of Medicine. We reviewed all therapeutic leukapheresis procedures performed in the apheresis unit between March 2012 and June 2026.

Adult patients (≥18 years) with a hematologic malignancy who underwent leukapheresis were eligible for inclusion. Procedures were excluded if pre- or post-procedure complete blood count data were unavailable, if the patient was younger than 18 years, or if the procedure was a non-leukapheresis apheresis modality (e.g., plasmapheresis, plateletpheresis). After applying these criteria, 125 procedures were included in the final analysis. Where a patient underwent more than one procedure, each procedure was treated as an independent analytical unit, consistent with the procedure-level focus of the study.

At our institution, therapeutic leukapheresis is initiated promptly upon presentation in patients with hyperleukocytosis, before the initiation of pharmacological cytoreductive therapy. Cytoreductive treatment—including hydroxyurea and disease-specific induction chemotherapy, together with allopurinol for tumor lysis prophylaxis—is generally started after the initial leukapheresis session, once the immediate risk of tumor lysis has been reduced. Consequently, all first procedures in this cohort were performed in a treatment-naïve state. Any pharmacological cytoreduction administered before a subsequent procedure was recorded, and all patients received standard intravenous hydration prior to the procedure.

### 2.2. Data Collection

Demographic, clinical, laboratory, and procedural data were retrospectively extracted from apheresis unit records and hospital medical charts. Collected variables comprised: patient age, sex, and diagnosis; pre- and post-procedure complete blood count parameters (WBC, hemoglobin, hematocrit, platelet count); pre- and post-procedure biochemical parameters (lactate dehydrogenase [LDH], creatinine, uric acid); and the presence or absence of clinical leukostasis. Procedural parameters included procedure duration, total processed blood volume, anticoagulant (acid-citrate-dextrose) volume and ratio, inlet flow rate, product volume, vascular access type, device used, and red cell priming (the use of a red blood cell-primed extracorporeal circuit, applied when clinically indicated, for example in patients with anemia or low body weight).

Clinical leukostasis was defined as the presence of signs or symptoms attributable to impaired microcirculatory flow in the setting of hyperleukocytosis, in the absence of an alternative explanation. Organ involvement was categorized as neurological (e.g., altered mental status, focal deficits, headache), pulmonary (e.g., dyspnea, hypoxemia, diffuse infiltrates), retinal (e.g., visual disturbance, retinal hemorrhage), or renal (e.g., acute kidney injury). The diagnosis of leukostasis was made clinically by the treating hematology team.

Diagnoses were categorized as acute myeloid leukemia (AML), acute lymphoblastic leukemia (ALL), chronic myeloid leukemia (CML), and other hematologic malignancies (comprising chronic lymphocytic leukemia, non-Hodgkin lymphoma, myelodysplastic syndrome, and chronic myelomonocytic leukemia). For procedures initially recorded as “acute leukemia, not otherwise specified,” the underlying subtype (AML or ALL) was assigned on the basis of each patient’s immunophenotypic and cytogenetic data, in accordance with the World Health Organization classification [13]. As these patients were diagnosed and followed within our hematology clinic, this subtype information was available from the routine clinical record.

### 2.3. Leukapheresis Procedure

Procedures were performed using a continuous-flow cell separator. The Spectra Optia system (Terumo BCT, Lakewood, CO, USA) was used in 123 of the 125 procedures (98.4%), and a Haemonetics system (Haemonetics Corporation, Boston, MA, USA) was used in the remaining 2 procedures (1.6%). Acid-citrate-dextrose solution A was used for anticoagulation. Vascular access was obtained via a central venous catheter, a hemodialysis catheter, or a peripheral venous route, according to clinical circumstances and vascular availability. Red cell priming was applied when clinically indicated. Procedural parameters were set and adjusted by the treating apheresis team in accordance with institutional practice [14].

### 2.4. Endpoints

The primary endpoint was the WBC reduction rate, calculated as: WBC reduction (%) = [(pre-procedure WBC − post-procedure WBC)/pre-procedure WBC] × 100, treated as a continuous variable. Secondary endpoints were the achievement of ≥30% and ≥50% WBC reduction, analyzed as binary outcomes. The ≥30% threshold was selected in accordance with commonly applied definitions of leukapheresis efficacy [7]. Thirty-day all-cause mortality was recorded as a clinical outcome measure.

Leukapheresis was performed for one of three indications, as determined by the treating team: symptomatic clinical leukostasis; isolated hyperleukocytosis without overt leukostatic symptoms; or prophylactic cytoreduction before the initiation of induction chemotherapy. The ≥30% and ≥50% WBC reduction thresholds were selected as pragmatic secondary endpoints: the ≥30% threshold reflects a commonly applied definition of a clinically meaningful single-procedure response [7], while the ≥50% threshold was applied as a study-defined benchmark of a marked reduction in circulating tumor burden. Both were analyzed as binary outcomes to complement the continuous primary endpoint.

### 2.5. Statistical Analysis

Continuous variables are presented as median (interquartile range [IQR]) and categorical variables as frequency (percentage). The distribution of continuous variables was assessed prior to analysis, and non-parametric tests were applied accordingly. Associations between continuous variables and the WBC reduction rate were evaluated using Spearman rank correlation. Between-group comparisons were performed using the Mann–Whitney U test (two groups) or the Kruskal–Wallis test (more than two groups); categorical variables were compared using the chi-square test. Where the Kruskal–Wallis test was significant, pairwise comparisons were performed using the Dunn test with Bonferroni correction. Effect sizes were reported as epsilon-squared for the Kruskal–Wallis test.

Variables associated with the WBC reduction rate at *p* < 0.10 in univariate analysis, together with clinically relevant predictors, were entered into a multivariable linear regression model. Because some patients contributed more than one procedure, cluster-robust (sandwich) standard errors clustered by patient were used for the primary multivariable model to account for within-patient correlation. As a sensitivity analysis, a linear mixed-effects model with a random intercept for patient was also fitted, and the intraclass correlation coefficient (ICC) was calculated. The distributional assumptions of the linear model were assessed by inspection of residuals. A multivariable logistic regression model was constructed for the ≥50% reduction endpoint. The multivariable linear model of the continuous reduction rate was the primary inferential model; the logistic model was considered secondary and exploratory. Model performance was summarized using the coefficient of determination (R^2^) for linear regression and the pseudo-R^2^ for logistic regression. Discrimination of the logistic model was assessed using the area under the receiver operating characteristic curve (AUC), and calibration was assessed using the Hosmer–Lemeshow goodness-of-fit test. Odds ratios with 95% confidence intervals were reported as effect sizes. Multicollinearity was assessed using the variance inflation factor. Receiver operating characteristic (ROC) analysis was used to identify optimal cutoff values for continuous predictors, with the Youden index used to determine the optimal threshold. Pre- and post-procedure biochemical parameters were compared using the Wilcoxon signed-rank test. A two-sided *p*-value < 0.05 was considered statistically significant. Analyses were performed using Python version 3.11 (Python Software Foundation, Wilmington, DE, USA; packages pandas, SciPy, statsmodels, and scikit-learn) and IBM SPSS Statistics version 24 (IBM Corp., Armonk, NY, USA). No formal sample size calculation was performed given the retrospective design; all eligible procedures performed within the study period were included. To assess potential temporal changes over the study period, the WBC reduction rate was compared between procedures performed in 2012–2018 and 2019–2026, in order to verify that the estimated procedural determinants were not confounded by secular changes in practice over the study period, and its correlation with calendar year was examined. For paired biochemical parameters, the pattern of missing data was examined by comparing the WBC reduction rate between procedures with and without available paired values.

## 3. Results

### 3.1. Baseline Characteristics

A total of 125 leukapheresis procedures performed in adult patients with hematologic malignancies were analyzed. The median patient age was 61 years (IQR 45–74), and 75 procedures (60%) were performed in male patients. The most frequent underlying diagnosis was AML (69 procedures, 55%), followed by ALL (24, 19%), CML (18, 14%), and other hematologic malignancies (14, 11%). Clinical leukostasis was present in 32 procedures (26%). The median pre-procedure WBC count was 236 × 10^9^/L (IQR 181–353). Baseline patient, laboratory, and procedural characteristics are summarized in Table 1.

With regard to pre-procedure management, all 94 first procedures were performed in a treatment-naïve state, and a further 13 repeat procedures were also performed without prior pharmacological cytoreduction, so that 107 of the 125 procedures (86%) were treatment-naïve overall. Among the remaining procedures, hydroxyurea had been administered before 13 (10%), corticosteroids before 5 (4%), and induction chemotherapy before 3 (2%); in every case this pharmacological cytoreduction preceded a repeat rather than a first procedure. The indication for leukapheresis was isolated hyperleukocytosis in 79 procedures (63%), symptomatic leukostasis in 32 (26%), and prophylactic cytoreduction before chemotherapy in 14 (11%). Among the 32 procedures with clinical leukostasis, pulmonary involvement was most common (17 procedures), followed by neurological (14) and renal (7) involvement; no retinal involvement was observed.

Vascular access was most commonly obtained via a central venous catheter (96 procedures, 77%), followed by a hemodialysis catheter (16, 13%) and a peripheral venous route (13, 10%). The Spectra Optia system was used in 123 procedures (98.4%) and a Haemonetics system in 2 (1.6%).

### 3.2. Leukapheresis Efficacy

**Figure 1:**
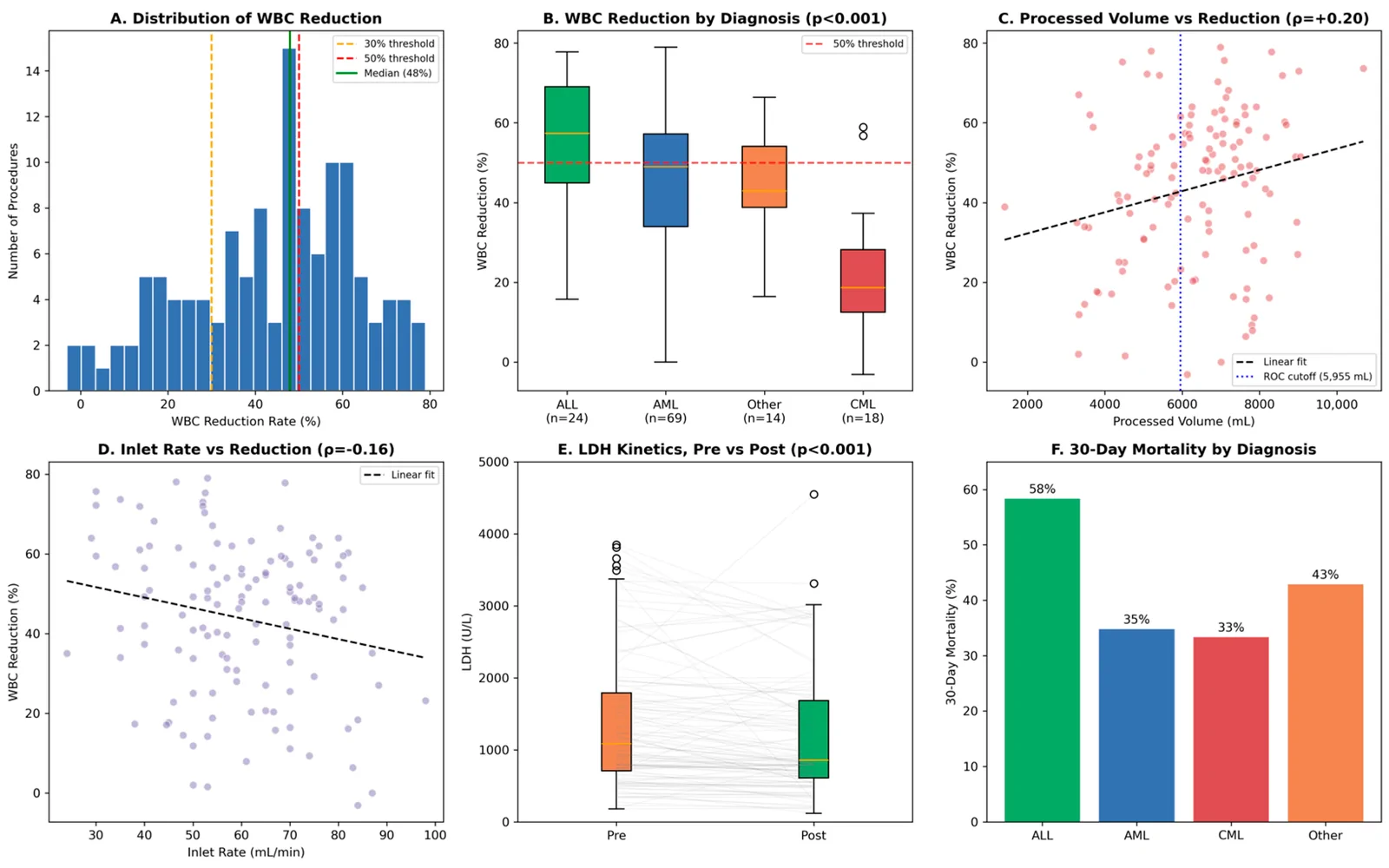
Leukapheresis efficacy and outcomes. (**A**) Distribution of WBC reduction rates across all procedures (dashed lines indicate the 30% and 50% reduction thresholds and the solid line the median); (**B**) WBC reduction by diagnostic group (the dashed line indicates the 50% threshold); (**C**) relationship between processed blood volume and WBC reduction, the dashed line is the linear fit and the dotted line the ROC-derived processed-volume cutoff; (**D**) relationship between inlet flow rate and WBC reduction (the dashed line is the linear fit); (**E**) LDH levels before and after leukapheresis; (**F**) 30-day mortality by diagnostic group.

Leukapheresis produced a significant reduction in the circulating leukocyte burden, with the median WBC count decreasing from 236 × 10^9^/L to 127 × 10^9^/L. The median WBC reduction rate was 47.9%. A reduction of ≥30% was achieved in 94 procedures (75%), and a reduction of ≥50% in 53 procedures (42%) (Figure 1A).

WBC reduction differed significantly across diagnostic groups (*p* < 0.001; Figure 1B). The greatest reduction was observed in ALL (median 57.4%, IQR 44.9–69.1), followed by AML (49.0%, IQR 34.0–57.3) and other malignancies (42.9%, IQR 38.8–54.1). The lowest reduction was observed in CML (18.6%, IQR 12.5–28.2). Consistent with this, acute leukemias collectively achieved substantially greater cytoreduction than chronic and other entities. Pairwise comparisons (Dunn test with Bonferroni correction) confirmed that this difference was driven by CML, which differed significantly from ALL (*p* < 0.001), AML (*p* < 0.001), and other malignancies (*p* = 0.018), whereas ALL and AML did not differ significantly from each other (*p* = 0.22). The effect size for the overall between-group difference was large (epsilon-squared = 0.20). By diagnostic group, the median post-procedure WBC count was 118 × 10^9^/L in ALL, 107 × 10^9^/L in AML, 187 × 10^9^/L in other malignancies, and 298 × 10^9^/L in CML, the last remaining well above the hyperleukocytosis range despite the procedure.

### 3.3. Univariate Predictors of WBC Reduction

In univariate analysis, processed blood volume (ρ = +0.20, *p* = 0.025) and total processed blood volume relative to patient blood volume (ρ = +0.26, *p* = 0.004) correlated positively with WBC reduction, whereas baseline platelet count (ρ = −0.26, *p* = 0.004), baseline creatinine (ρ = −0.22, *p* = 0.014), and inlet flow rate (ρ = −0.16, *p* = 0.081) showed negative associations (Table 2). Baseline WBC count, LDH, uric acid, age, and procedure duration were not significantly associated with the reduction rate. Neither vascular access type (*p* = 0.275) nor the presence of clinical leukostasis (*p* = 0.534) was significantly associated with the WBC reduction rate.

### 3.4. Multivariable Analysis

In the multivariable linear regression model, four variables were independently associated with the WBC reduction rate, with standard errors clustered by patient (R^2^ = 0.27, model *p* < 0.001; Table 3): processed volume (β = 0.004, 95% CI 0.002 to 0.006, *p* < 0.001), inlet flow rate (β = −0.43, 95% CI −0.62 to −0.24, *p* < 0.001), acute leukemia diagnosis (β = 10.4, 95% CI 0.5 to 20.4, *p* = 0.040), and baseline platelet count (β = −0.03, 95% CI −0.06 to −0.00, *p* = 0.036). A higher processed volume and an acute leukemia diagnosis were associated with greater WBC reduction, whereas a higher inlet flow rate and a higher baseline platelet count were associated with lesser reduction. No relevant multicollinearity was detected. A linear mixed-effects model with a random intercept for patient yielded consistent results (intraclass correlation coefficient 0.39), confirming that the associations were robust to within-patient clustering.

In the logistic regression model for ≥50% reduction, processed volume (OR 1.001 per mL, *p* = 0.002) and inlet flow rate (OR 0.96, *p* = 0.013) remained independent predictors, with 95% confidence intervals of 1.0002–1.0008 for processed volume and 0.93–0.99 for inlet flow rate. The model showed acceptable discrimination (area under the ROC curve 0.68) and adequate calibration (Hosmer–Lemeshow *p* = 0.23). ROC analysis identified an optimal processed-volume cutoff of approximately 5955 mL for achieving ≥50% reduction (Figure 1C).

### 3.5. Biochemical Kinetics

Leukapheresis was associated with significant reductions in biochemical markers of tumor burden. Median LDH decreased from 1085.5 to 854.0 U/L (*p* < 0.001), and median uric acid decreased from 7.5 to 5.6 mg/dL (*p* < 0.001). Creatinine showed a smaller but statistically significant decrease (*p* = 0.015) (Table 4, Figure 1E).

Paired biochemical data were available for 118 procedures for LDH (94%), 109 for uric acid (87%), and 123 for creatinine (98%). The WBC reduction rate did not differ between procedures with and without available paired LDH (*p* = 0.65) or uric acid (*p* = 0.23) values, consistent with data missing at random, although missingness was somewhat more frequent in CML, in which tumor lysis monitoring was less often performed. Hematocrit did not change significantly between the pre- and post-procedure measurements (median 24.0% to 24.0%, *p* = 0.55), indicating that the observed biochemical reductions were not attributable to hemodilution.

### 3.6. Thirty-Day Mortality

Overall 30-day all-cause mortality was 40% (50 procedures). Mortality varied across diagnostic groups, being highest in ALL (58%) and other malignancies (43%), and lower in AML (35%) and CML (33%) (Figure 1F). Notably, achievement of ≥50% WBC reduction was not associated with lower 30-day mortality; rather, mortality tended to be higher among procedures achieving greater cytoreduction, likely reflecting the preferential use of aggressive leukapheresis in patients with the highest disease burden. This exploratory finding should not be interpreted as evidence that leukapheresis worsens outcomes; rather, it reflects confounding by indication.

Over the study period, the WBC reduction rate did not change significantly: the median reduction was 48.1% in procedures performed during 2012–2018 and 47.9% during 2019–2026 (*p* = 0.35), with no correlation between calendar year and reduction (ρ = −0.07, *p* = 0.45). This suggests that procedural efficacy remained stable despite the long inclusion period.

### 3.7. Sensitivity Analysis

To address the potential within-patient correlation arising from patients who underwent more than one procedure, we repeated the principal analyses using only the first procedure from each patient (*n* = 94). The results were essentially unchanged. The median WBC reduction was 48.2% (versus 47.9% in the full cohort), and the difference across diagnostic groups remained highly significant (*p* < 0.001), with the same ordering (ALL 56.6%, AML 49.3%, other 44.2%, CML 18.6%). In the multivariable linear regression model (*n* = 94, R^2^ = 0.29), processed volume (β = 0.004, *p* < 0.001), inlet flow rate (β = −0.39, *p* = 0.001), and acute leukemia diagnosis (β = 9.49, *p* = 0.040) remained independent predictors, while baseline platelet count showed a consistent direction with borderline significance (β = −0.033, *p* = 0.058). These findings indicate that the main results are robust to the inclusion of repeated procedures (Table 5). The two procedures separated from a prior procedure by more than 30 days (631 and 36 days) represented distinct clinical episodes; treating them as independent did not alter the results.

## 4. Discussion

In this retrospective single-center analysis of 125 leukapheresis procedures, we found that the efficacy of therapeutic leukapheresis—measured as the WBC reduction rate—was determined by both disease biology and modifiable procedural parameters. The median reduction of 47.9% is consistent with the range reported in previous series [15,16,17]. Our principal findings are threefold: efficacy differed markedly by disease subtype, being highest in acute leukemias and lowest in CML; processed blood volume and inlet flow rate were independent, potentially modifiable procedural determinants; and leukapheresis produced measurable reductions in biochemical markers of tumor burden.

The most pronounced finding was the disparity in cytoreduction across diagnostic groups. Acute lymphoblastic and myeloid leukemias achieved median reductions of 57.4% and 49.0%, respectively, whereas CML achieved only 18.6%. This difference persisted as an independent predictor in multivariable analysis. Several biological factors may account for the poor response in CML. In our cohort, CML procedures were characterized by a substantially higher baseline WBC count (median 361 vs. 224 × 10^9^/L, *p* < 0.001) and a markedly higher platelet count (154 vs. 47 × 10^9^/L, *p* < 0.001) compared with acute leukemias. The very high circulating cell mass in CML, combined with the different physical and rheological properties of the myeloid cell population, likely limits the fractional reduction achievable in a single procedure [5,15]. In addition, the lower processed volume in CML procedures (median 5137 vs. 6603 mL, *p* = 0.064) may have further contributed to the reduced efficacy, highlighting the interplay between disease biology and procedural intensity [18]. Taken together, these observations suggest that patients with CML may require repeated procedures to achieve adequate cytoreduction.

Among procedural parameters, processed blood volume emerged as a robust independent predictor of greater WBC reduction in both linear and logistic models, with ROC analysis identifying an approximate threshold of 5955 mL for achieving ≥50% reduction; this cutoff is derived from a single center and requires external validation before it can be generalized. This dose–response association is potentially actionable, suggesting that processing a larger blood volume, where hemodynamically tolerated, may be associated with greater cytoreduction [19]. Conversely, a higher inlet flow rate was independently associated with lesser reduction (Figure 1D), which may reflect reduced separation efficiency at higher flow rates [11]. Importantly, the baseline platelet count was inversely associated with reduction, consistent with the negative association observed for CML, in which thrombocytosis is common. Notably, neither vascular access type nor the presence of clinical leukostasis influenced efficacy; the former finding is reassuring, indicating that peripheral venous access, when feasible, does not appear to compromise procedural effectiveness [20], although this comparison involved a small number of peripheral-access procedures and may be underpowered to detect a modest difference.

The observed reductions in LDH and uric acid following leukapheresis are of particular interest. Both are established markers of tumor cell turnover and burden, and their decline is consistent with a reduction in the overall metabolic tumor load, potentially mitigating the risk of tumor lysis syndrome during subsequent induction chemotherapy [21]. Prior work has identified elevated LDH and creatinine as risk factors for early death in hyperleukocytic acute leukemia [22]. While our study was not designed to assess tumor lysis prophylaxis directly, these biochemical kinetics provide a mechanistic rationale that merits prospective investigation. Because first procedures were performed before any cytoreductive therapy and no patient received rasburicase, and because hematocrit did not change across the procedure, these reductions are unlikely to be explained by concurrent pharmacological treatment or hemodilution; a contribution from regression to the mean cannot, however, be excluded in this uncontrolled paired design. Moreover, the short duration of the procedure (median 113 min) makes a pharmacological contribution to the observed intra-procedural changes unlikely: agents such as hydroxyurea lower the circulating leukocyte count over 24–48 h through inhibition of cell proliferation, a timeframe far exceeding the interval between the pre- and post-procedure measurements.

An exploratory observation was that achievement of ≥50% WBC reduction was not associated with lower 30-day mortality; if anything, mortality was numerically higher among procedures achieving greater cytoreduction. This association should be interpreted with considerable caution. It emerged solely from an exploratory analysis and was not a predefined aim of the study, which was designed to characterize the technical efficacy of leukapheresis rather than its effect on survival. Thirty-day mortality in this population is influenced by numerous factors—including disease subtype, cytogenetic and molecular risk, performance status, comorbidities, infectious complications, and response to induction chemotherapy—none of which were incorporated into this analysis. All patients received standard disease-directed treatment in addition to leukapheresis, and we did not examine post-procedure clinical follow-up parameters. The most plausible explanation for the observed pattern is confounding by indication: patients with the highest disease burden and most severe presentation—who carry the worst baseline prognosis—are precisely those subjected to the most aggressive leukapheresis [23]. Accordingly, these data underscore the conceptual distinction between the technical efficacy of leukapheresis (its ability to lower the WBC count) and its clinical benefit (its effect on survival); the latter cannot be inferred from our findings and remains a subject of ongoing debate in the absence of randomized evidence [8,24,25]. The overall 30-day mortality of 40% in our cohort is consistent with the high early mortality reported in hyperleukocytic hematologic malignancies [3,12].

This study has several strengths. All patients were diagnosed and managed within our hematology clinic, ensuring that diagnostic subtypes were accurately established from complete clinical, immunophenotypic, and cytogenetic records rather than from apheresis logs alone. Further strengths include the relatively large procedure count for a single-center leukapheresis series, the comprehensive capture of procedural and device parameters that are frequently absent from prior reports, and the inclusion of paired pre- and post-procedure biochemical data.

This study has several limitations. First, its retrospective, single-center design carries an inherent risk of selection bias and limits generalizability. Second, procedural decisions were made at the discretion of the treating team rather than by protocol, introducing the confounding by indication discussed above. Third, the study period spanned more than a decade, during which practice patterns may have evolved. Fourth, the multivariable model explained a modest proportion of the variance in WBC reduction (R^2^ = 0.27), indicating that unmeasured factors also contribute to efficacy. Fifth, some patients contributed more than one procedure; we addressed the resulting within-patient correlation using cluster-robust standard errors and a mixed-effects model, and a sensitivity analysis restricted to first procedures yielded essentially identical results, supporting the robustness of our findings. Sixth, several clinically important prognostic variables—including performance status, cytogenetic and molecular risk, and disease burden beyond the WBC count—were not available and could not be incorporated, so residual confounding cannot be excluded. Finally, clinical outcomes such as resolution of leukostasis, organ failure, and confounder-adjusted survival were beyond the scope of this study, which focused on technical cytoreduction, and represent an important direction for future work. These limitations should be addressed in future prospective, multicenter studies.

## 5. Conclusions

In this single-center cohort, the efficacy of therapeutic leukapheresis was associated with both disease subtype and modifiable procedural parameters. Acute leukemias responded substantially better than CML, and a higher processed blood volume together with a lower inlet flow rate were independently associated with greater leukocyte reduction. Leukapheresis additionally reduced biochemical markers of tumor burden. These findings suggest that procedural optimization—particularly maximizing processed volume where clinically tolerated—may be associated with greater cytoreduction, while the poor response of CML warrants tempered expectations, as reflected in the limited single-procedure reduction observed, and may require repeated procedures to achieve adequate cytoreduction. The exploratory finding of no association between cytoreduction and short-term mortality, which the present design cannot assess robustly, highlights the distinction between technical efficacy and clinical benefit, a question that should be addressed in prospective, multicenter studies.

## Figures and Tables

**Table 1:** Baseline patient, laboratory, and procedural characteristics.

Characteristic	Value (*n* = 125)
Age, years, median (IQR)	61 (45–74)
Male sex, *n* (%)	75 (60)
AML, *n* (%)	69 (55)
ALL, *n* (%)	24 (19)
CML, *n* (%)	18 (14)
Other, *n* (%)	14 (11)
Pre-WBC, ×10^9^/L	236 (181–353)
Pre-hemoglobin, g/dL	8.0 (7.0–9.4)
Pre-platelet, ×10^9^/L	51 (30–84)
Pre-LDH, U/L	1073 (669–1747)
Pre-creatinine, mg/dL	1.1 (0.8–1.6)
Pre-uric acid, mg/dL	7.5 (4.8–11.2)
Leukostasis present, *n* (%)	32 (26)
Procedure duration, min	113 (103–126)
Processed volume, mL	6628 (5200–7608)
Inlet rate, mL/min	60 (50–70)
Central venous catheter, *n* (%)	96 (77)
Hemodialysis catheter, *n* (%)	16 (13)
Peripheral venous, *n* (%)	13 (10)

Continuous variables are presented as median (interquartile range [IQR]); categorical variables as *n* (%).

**Table 2:** Univariate correlation of continuous variables with WBC reduction rate.

Variable	Spearman ρ	*p*-Value
Age	+0.001	0.987
Pre-WBC	−0.079	0.384
Pre-platelet	−0.256	0.004
Pre-LDH	+0.115	0.209
Pre-creatinine	−0.220	0.014
Pre-uric acid	−0.139	0.140
Procedure duration	+0.100	0.266
Processed volume	+0.201	0.025
Total processed volume/PBV	+0.258	0.004
Inlet rate	−0.157	0.081

PBV, patient blood volume. Spearman rank correlation.

**Table 3:** Multivariable linear regression for WBC reduction rate.

Variable	β	95% CI	*p*-Value
Pre-platelet	−0.031	−0.059 to −0.002	0.036
Processed volume	0.004	0.002 to 0.006	<0.001
Acute leukemia	10.43	0.50 to 20.36	0.040
Inlet rate	−0.426	−0.616 to −0.235	<0.001

R^2^ = 0.27, adjusted R^2^ = 0.25, model *p* < 0.001. *p*-values and confidence intervals are based on standard errors clustered by patient.

**Table 4:** Biochemical parameter kinetics before and after leukapheresis.

Parameter	Pre (Median)	Post (Median)	*p*-Value	*n*
LDH, U/L	1085.5	854.0	<0.001	118
Creatinine, mg/dL	1.1	1.1	0.015	123
Uric acid, mg/dL	7.5	5.6	<0.001	109

Wilcoxon signed-rank test for paired samples.

**Table 5:** Comparison of multivariable predictors between the full cohort and the first-procedure-only sensitivity analysis.

Predictor	Full Cohort β (*p*)	First-Procedure β (*p*)
Processed volume	0.004 (<0.001)	0.004 (<0.001)
Inlet flow rate	−0.43 (<0.001)	−0.39 (<0.001)
Acute leukemia	10.43 (0.040)	9.49 (0.040)
Baseline platelet	−0.031 (0.036)	−0.033 (0.058)

β, regression coefficient. Full cohort *n* = 125; first-procedure-only cohort *n* = 94.

## Data Availability

The data presented in this study are available on request from the corresponding author. The data are not publicly available due to privacy restrictions.

## Abbreviations

The following abbreviations are used in this manuscript:

WBC	White blood cell
AML	Acute myeloid leukemia
ALL	Acute lymphoblastic leukemia
CML	Chronic myeloid leukemia
LDH	Lactate dehydrogenase
IQR	Interquartile range
ROC	Receiver operating characteristic
ASFA	American Society for Apheresis
